# Supplementary material for: Symptomatic dry eye disease (DED) in cohort of contact lens wearers in Jordan
Source: PLoS One. 2025 Oct 27;20(10):e0335254. doi: 10.1371/journal.pone.0335254 (PMC12558516; doi:10.1371/journal.pone.0335254)
Supplement: S2 File — (DOCX) [file pone.0335254.s002.docx]

**Table S1: Test of Normality – Shapiro-Wilk test.**

|  | Statistic | Df | p-value |
| --- | --- | --- | --- |
| OSDI_Score | 0.946 | 301 | 0.081 |

**Table S2: Test of homogeneity – Levene’s test.**

|  |  | Levene Statistic | Df1 | Df2 | Sig. |
| --- | --- | --- | --- | --- | --- |
| Gender | Based on Mean | 0.379 | 1 | 299 | 0.538 |
|  | Based on Median | 0.391 |  |  | 0.532 |
|  | Based on trimmed Mean | 0.408 |  |  | 0.523 |
| Age | Based on Mean | 4.912 | 2 | 298 | 0.180 |
|  | Based on Median | 4.343 |  |  | 0.141 |
|  | Based on trimmed Mean | 4.666 |  |  | 0.101 |
| Contact Lens Type | Based on Mean | 0.979 | 1 | 298 | 0.323 |
|  | Based on Median | 0.901 |  |  | 0.343 |
|  | Based on trimmed Mean | 0.920 |  |  | 0.338 |
| CL use daily | Based on Mean | 1.878 | 2 | 297 | 0.155 |
|  | Based on Median | 1.754 |  |  | 0.175 |
|  | Based on trimmed Mean | 1.849 |  |  | 0.159 |
| Contact lens age | Based on Mean | 2.409 | 2 | 298 | 0.071 |
|  | Based on Median | 2.412 |  |  | 0.079 |
|  | Based on trimmed Mean | 2.438 |  |  | 0.083 |
| Frequency of lubricant eye drops use | Based on Mean | 1.242 | 2 | 297 | 0.147 |
|  | Based on Median | 1.217 |  |  | 0.138 |
|  | Based on trimmed Mean | 1.254 |  |  | 0.142 |
| Cleaning rate of CL | Based on Mean | 1.015 | 2 | 297 | 0.110 |
|  | Based on Median | 0.935 |  |  | 0.121 |
|  | Based on trimmed Mean | 1.089 |  |  | 0.103 |
| Usage of mask during CL usage | Based on Mean | 3.484 | 1 | 299 |  |
|  | Based on Median | 2.633 |  |  |  |
|  | Based on trimmed Mean | 3.381 |  |  |  |
| Frequency of mask use per day | Based on Mean | 0.809 | 2 | 298 | 0.421 |
|  | Based on Median | 0.820 |  |  | 0.398 |
|  | Based on trimmed Mean | 0.829 |  |  | 0.411 |
|  |  |  |  |  |  |

**Table S3 Multiple regression analysis results**

| **Model Summary** | | | | | |
| --- | --- | --- | --- | --- | --- |
| **R-square** | 0.828 | | **Adjusted R-square** | | 0.821 |
| **ANOVA Summary** | | | | | |
| **F-value** | 126.245 | | **P-value** | | <0.001 |
| **Coefficients** | | | | | |
|  | **Coefficients** | | **Significance** | **Collinearity Statistics** | |
|  | **Unstandardized B** | **Standardized Beta** | **P-value** | **Tolerance** | **VIF** |
| **Constant** | 20.539 | --- | <0.001 | --- | --- |
| **Age** | -0.433 | -0.014 | 0.572 | 0.906 | 1.104 |
| **Gender** | 0.895 | 0.022 | 0.394 | 0.894 | 1.119 |
| **CL_Mask** | -0.909 | -0.026 | 0.305 | 0.898 | 1.113 |
| **CL_use_year** | 2.466 | 0.112 | <0.001 | 0.708 | 1.413 |
| **Do_you_use_mask** | 0.611 | 0.018 | 0.730 | 0.224 | 4.472 |
| **Mask_use_freq.** | 9.745 | 0.459 | <0.001 | 0.201 | 4.973 |
| **CL_symptoms** | 2.234 | 0.124 | <0.001 | 0.808 | 1.237 |
| **CL_types** | -0.064 | -0.001 | 0.959 | 0.889 | 1.125 |
| **CL_use_daily** | -0.189 | -0.007 | 0.778 | 0.857 | 1.167 |
| **CL_cleaning_rate** | -4.445 | -0.214 | <0.001 | 0.339 | 2.949 |
| **Eyedrop_use** | -4.430 | -0.209 | <0.001 | 0.326 | 3.067 |


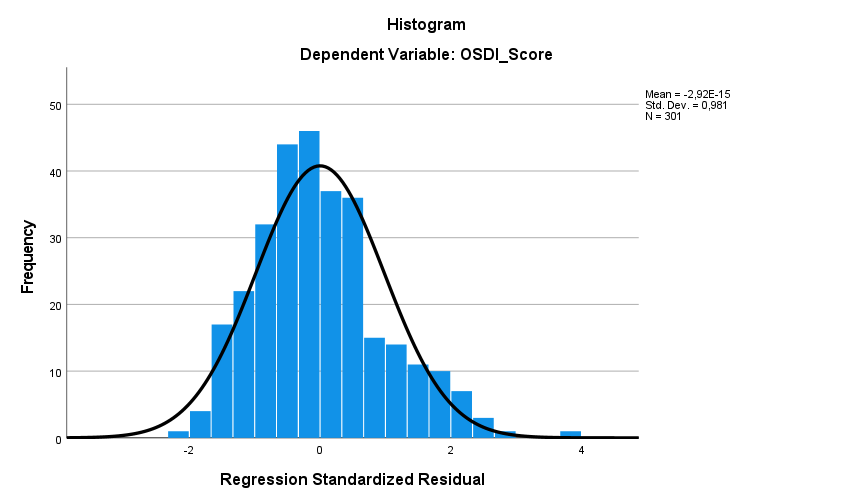


**Figure S1: Regression Standardized Residual plot**


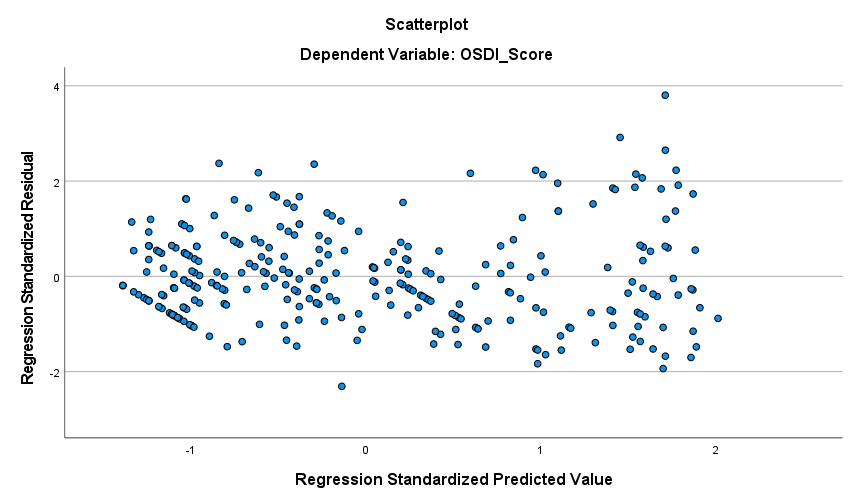


**Figure S2: Regression Standardized Predicted value scatter plot**
